# Overexpression of Long Non-Coding RNA MIR22HG Represses Proliferation and Enhances Apoptosis via miR-629-5p/TET3 Axis in Osteosarcoma Cells

**DOI:** 10.4014/jmb.2106.06028

**Published:** 2021-08-09

**Authors:** Haoliang Zhao, Ming Zhang, Xuejing Yang, Dong Song

**Affiliations:** 1 Orthopedics Department, Shanxi Bethune Hospital, Shanxi Academy of Medical Sciences, Taiyuan City, Shanxi Province 030032, P.R. China; 2 Cancer Center, Shanxi Bethune Hospital, Shanxi Academy of Medical Sciences, No. 99 Longcheng Street, Xiaodian District, Taiyuan City, Shanxi Province 030032, P.R. China

**Keywords:** Osteosarcoma, MIR22 host gene, microRNA-629-5p, tet methylcytosine dioxygenase 3, proliferation, apoptosis

## Abstract

In this study, we evaluated the mechanism of long non-coding RNA MIR22 host gene (LncRNA MIR22HG) in osteosarcoma cells. Forty-eight paired osteosarcoma and adjacent tissues samples were collected and the bioinformatic analyses were performed. Target genes and potential binding sites of MIR22HG, microRNA (miR)-629-5p and tet methylcytosine dioxygenase 3 (TET3) were predicted by Starbase and TargetScan V7.2 and confirmed by dual-luciferase reporter assay. Cell Counting Kit-8, colony formation and flow cytometry assays were utilized to determine the viability, proliferation and apoptosis of transfected osteosarcoma cells. Pearson’s analysis was introduced for the correlation analysis between MIR22HG and miR-629-5p in osteosarcoma tissue. Relative expressions of MIR22HG, miR-629-5p and TET3 were measured by quantitative real-time polymerase chain reaction or Western blot. MiR-629-5p could competitively bind with and was negatively correlated with MIR22HG, the latter of which was evidenced by the high expression of miR-629-5p and low expression of MIR22HG in osteosarcoma tissues. Overexpressed MIR22HG repressed the viability and proliferation but enhanced apoptosis of osteosarcoma cells, which was reversed by miR-629-5p upregulation. TET3 was the target gene of miR-629-5p, and the promotive effects of upregulated miR-629-5p on the viability and proliferation as well as its repressive effect on apoptosis were abrogated via overexpressed TET3. To sum up, overexpressed MIR22HG inhibits the viability and proliferation of osteosarcoma cells, which was achieved via regulation of the miR-629-5p/TET3 axis.

## Introduction

Osteosarcoma, a common malignant bone tumor, has become recognized as the most prevalent primary bone cancer in children and adolescents, and the third most frequent bone cancer in adults [7]. The knee joint is the most frequently affected area and onset of the disease is preceded by mutation of the bone cells [20]. Despite great progress having been made in current clinical therapy for osteosarcoma including wide tumor excision, radiotherapy, and adjuvant chemotherapy, the prognosis for most patients remains unsatisfactory due to the high rate of metastasis and recurrence [36, 37]. Consequently, the development of a new clinical cure for patients diagnosed with osteosarcoma is a matter of great significance.

Long non-coding RNAs (lncRNAs), once considered as “evolutionary junk,” are now categorized as RNAs over 200 nucleotides in length without protein-translation capacity [1, 3]. A growing amount of research has revealed that lncRNAs play important regulatory roles in various pathophysiological processes, and multiple lncRNAs are reported to be dysregulated in several types of cancers such as osteosarcoma [12, 27, 38]. LncRNA HOX Transcript Antisense RNA (HOTAIR) represses mineralization in osteoblastic osteosarcoma cells via repressing ALPL [23]. LncRNA E2F1-regulated Inhibitor of Cell Death (ERICD) can interact with AT-rich interaction domain 3A (ARID3A) via the E2F transcription factor 1 (E2F1) so as to regulate the proliferation and migration of osteosarcoma cells [4]. In addition, lncRNA p21-associated ncRNA DNA damage-activated (PANDA) has also been implicated in the proliferation of osteosarcoma cells [17]. As for MIR22 host gene (MIR22HG), a previous publication has provided the evidence concerning its participation in promoting the osteogenic differentiation of bone marrow mesenchymal stem cells (BM-MSCs) [14]. However, so far as we are concerned, there’s inadequate discussion on its role in osteosarcoma, which is thus reported in this study.

In addition, mounting evidence has also demonstrated the participation of microRNAs (miRNAs, miRs) in osteosarcoma [24]. MiRNAs are members of non-coding RNAs (ncRNAs) which can modulate gene expression at a post-transcriptional level via suppression of messenger RNA (mRNA) translation or via promotion of mRNA degradation [6]. MiR-193 has been identified as one of the small molecular silencers in the therapy for osteosarcoma [13]. Also, miR-21 is a critical, major player in the pathogenesis and progression of osteosarcoma [28]. In addition, the role of miR-142 as a suppressor in the pathogenesis of osteosarcoma has been well discussed, along with the interpretation of its potential as a therapeutic approach for osteosarcoma [29]. Furthermore, the elevation of miR-629 in osteosarcoma has been shown and miR-629 promotes the cell proliferation, migration and invasion of osteosarcoma and its expression can be used to predict the prognosis of patients [18]. Nevertheless, the interaction between miR-629-5p and lncRNAs, MIR22HG in particular, has not been elucidated yet. Thus, we hereby report the regulatory functions of both LncRNA MIR22HG and miRNA miR-629-5p in osteosarcoma, hoping to provide new evidence concerning the implication of MIR22HG in osteosarcoma.

## Materials and Methods

### Ethical Statement

The conduction of our study has been ethically approved by the Ethics Committee of Shanxi Bethune Hospital Shanxi Academy Of Medical Sciences (Approval Serial No. G2019020601K), and all volunteering patients or their legitimate guardians have provided written informed consent, agreeing to the usage for their tissues for research.

### Bioinformatic Analysis

To confirm that miR-629-5p could competitively bind with MIR22HG, the data concerning the complementary binding sites between MIR22HG and miR-629-5p were available from Starbase (http://starbase.sysu.edu.cn/). Meanwhile, for the prediction of target genes, we firstly downloaded the data from both Starbase and TargetScan V7.2 (http://www.targetscan.org/vert_72/), and retrieved the dataset GSE70415 (the mRNA and miRNA expression from human osteosarcoma) from GEO (https://www.ncbi.nlm.nih.gov/geo/). All the results were finally summarized and displayed in a Venn diagram which was drawn and downloaded from Venny online software V2.1.0 (http://bioinfogp.cnb.csic.es/tools/venny/). Six common mRNAs were displayed, among which tet methylcytosine dioxygenase 3 (TET3) aroused our interest, as it has been shown to be the target gene of miRNA and participate in the development and progression of osteosarcoma [15].

### Clinical Specimen

Clinical specimens of osteosarcoma tissues (Cancer tissue, *n* = 48) and paired adjacent tissues (Adjacent tissue, *n* = 48) of patients diagnosed with osteosarcoma were collected from Shanxi Bethune Hospital Shanxi Academy of Medical Sciences between 2019 February and 2020 January. The osteosarcoma tissue was obtained by biopsy for diagnosis, and the paired adjacent tissue was collected during surgical operation. All of the patients conformed with the following criteria: a) patients had not received any treatment, chemotherapy or radiotherapy, for instance; b) patients were not diagnosed with other cancers, autoimmune diseases or infectious diseases, etc. All samples were well preserved at -80°C after being washed using phosphate-buffered saline (PBS, P4417, Sigma-Aldrich, USA).

### Cell Culture and Transfection

Osteosarcoma cell line MG63 (iCell-h140) was bought from iCell (China), while SOSP-9607, another osteosarcoma cell line, was obtained from the Department of Orthopedic Surgery, Tangdu Hospital, Fourth Military Medical University (Xi’an, China). Then, MG63 cells were cultured in minimal essential medium (MEM, PM150410, Procell, China), while RPMI-1640 medium (PM150110, Procell) was used for the culture of SOSP-9607 cells. All media were blended with 10% fetal bovine serum (FBS, 164210, Procell) and 1% penicillin-streptomycin (PB180120, Procell), and all cells were incubated in a humidified incubator (51030301, ThermoFisher Scientific, USA) at 37°C with 5% CO_2_.

Small interfering RNAs for both MIR22HG and TET3 (si-MIR22HG and siTET3) as well as their controls (si-control and siNC), miR-629-5p mimic (mimic), and inhibitor along with their controls (mimic NC and inhibitor NC), were all bought from Gene Pharma (China). Overexpressions of both MIR22HG and TET3 were constructed by inserting the sequence of MIR22HG and TET3 into the pcDNA 3.1 plasmid (V790-20, Invitrogen, USA), and the empty plasmid without insertion was used as the control (control for MIR22HG) or NC (control for TET3). The sequences for the transfection are listed in Table 1.

MG63 and SOSP-9607 cells (1 × 10^6^ cells/well) were cultured in 6-well plates at room temperature (RT) with the complete medium to be 90% confluent at the time point of transfection, and the transfection of both cells was performed with Lipofectamine (LFN) 2000 transfection reagent (11668-030, Invitrogen) after dilution with Opti-MEM reduced serum medium (22600-050, Gibco, USA). After 48 h, all cells were harvested for subsequent studies.

### Dual-Luciferase Reporter Assay

The wild-type (wt) or mutated (mut) sequences of MIR22HG and TET3 containing the binding or targeting sites of miR-629-5p or not were designed by Gene Pharma (China) and inserted into the dual-luciferase reporter vector pmirGLO (VT1439, Youbio, China), which were subsequently designated as the reporter plasmids of MIR22HG-wt (sequence: 5’-GAAAGUGAUACUAAACCCA-3’), MIR22HG-mut (sequence: 5’-GAAAAGUGA UACUGGGCCCA-3’), TET3-wt (sequence: 5’-UAAUUUCAUUGAGAGGGACCCAG-3’) and TET3-mut (sequence: 5’-UAAUUUCAUUGAGAGAAACCCAG-3’).

Prior to the dual-luciferase reporter assay, we adjusted the density of MG63 and SOSP-9607 cells in the 48-well plates to 3 × 10^5^ cells/well, and the reporter plasmids were transfected into these two cells along with miR-629-5p mimic or mimic NC using LFN 2000 transfection reagent at RT as instructed by the producer, followed by harvest and detection of the luciferase activity using the dual-luciferase report assay system (E1910, Promega, USA) and a Synergy HT Multi-Mode Microplate Reader (BioTek Instruments, Inc., USA) in accordance with the manufacturers' protocols. The luciferase activity was finally normalized to *Renilla* luciferase activity.

### Cell Viability, Proliferation and Apoptosis Assays

The Cell Counting Kit-8 (CCK-8) assay (CCK-8 Assay Kit CK04, Dojindo, Japan) was used to detect the viability of the transfected osteosarcoma cells MG63 and SOSP-9607. MG63 and SOSP-9607 cells (2 × 10^3^ cells/well) were cultured in a 96-well plate at 37°C with 5% CO_2_ for 24, 48, and 72 h. Ten microliters of CCK-8 solution was added to the plates, which were further incubated for 4 h to detect the cell viability. The optical density (OD) values at an absorbance of 450 nm were measured by a microplate reader (Model 680, Bio-Rad Laboratories, Inc., USA), and the viability of the transfected cells was calculated with the following formula: cell viability (%) = [OD (dosage)-OD (blank)]/[OD (no dosage)-OD (blank)] × 100%, where OD (dosage) represents the OD value of the wells with transfected cells and CCK-8 reagent, OD (blank) is the OD value of the wells with the medium and CCK-8 reagent only, and OD (no dosage) stands for the OD value of the wells that were added with untransfected cells, which were confirmed in the manufacturer's manual.

To determine the proliferation of transfected osteosarcoma cells MG63 and SOSP-9607, the colony formation assay was adopted. Cells (1 × 10^3^ cells/well) were cultured in a 6-well plate at 37°C with 5% CO_2_. After two weeks, cells were fixed with methanol (A506806, Sangon Biotech, China) for 15 min and stained with crystal violet (A100528, Sangon Biotech) for 30 min. Photos of visible colonies formed were taken with a digital camera (D500, Nikon, Japan), and the colony number was calculated with SigmaPlot (v. 11, Systat Software Inc., USA).

Flow cytometry was adopted to detect the apoptosis of transfected osteosarcoma cells MG63 and SOSP-9607 via an Annexin V-FITC cell apoptosis detection kit (E606336, Sangon Biotech). According to the manufacturer’s protocols, all the components of the kit, including the Annexin V-FITC, 4 × binding buffer and propidium iodide (PI), were thawed at RT before use. The 4 × binding buffer was diluted using 1 × distilled water, and all transfected cells were washed with phosphate-buffered saline (PBS, E607008, Sangon Biotech). Then, all cells were resuspended in 195 μl 1 × binding buffer at a density of 2 × 10^5^ cells/ml. Five microliters of Annexin V-FITC was added and cells were further incubated at RT for 15 min with no light, followed by washing in 200 μl 1 × binding buffer. After centrifugation at 1,000 ×*g* using a Megafuge 16R Centrifuge (75004270, ThermoFisher Scientific) for 5 min, the supernatant was discarded and all cells were resuspended in 190 μl 1 × binding buffer. Finally, 10 μl PI was added into cells, and the apoptosis was analyzed using an Attune NxT Flow Cytometer (A24864, ThermoFisher Scientific). All data were analyzed using Kaluza C Analysis Software (v. 1.1.2, Beckman Coulter, USA).

### RNA Isolation and Quantitative Real-Time Polymerase Chain Reaction (qRT-PCR)

Total RNA from tissues (osteosarcoma and adjacent) and cells (both transfected and untransfected) was extracted by Trizol reagent (B610409, Sangon Biotech) according to the manufacturer's manual and was then placed in a -80°C refrigerator. The concentration of total RNA was quantified using a biological spectrometer (Nano Drop 2000, ThermoFisher Scientific). After that, qRT-PCR was performed using a one-step qRT-PCR kit (D7277M, Beyotime, China) with the components as follows: 10 μl 2 × probe one-step reaction buffer, 2 μl 10 × probe one-step enzyme-mix, 2 μl mixture of both forward and reverse primer, 0.5 μl probe, 2 μl template RNA and RNase-free water (which was added up to 20 μl) within a CFX96 Touch Real-Time PCR Detection System (1855195, Bio-Rad Laboratories, Inc.). The experiment was conducted in the following conditions: 1) reverse transcription at 50°C for 15 min, 2) pre-denaturation at 95°C for 2 min, 3) denaturation at 95°C for 15 sec, and 4) annealing and extension at 60°C for 15 sec. The denaturation, annealing and final extension were repeated for 40 cycles in total. The primers were shown in Table 2. GAPDH (for MIR22HG and TET3) and U6 (for miR-629-5p) were used as internal references. The relative expressions were analyzed and quantified by 2^-ΔΔCT^ calculation method [19].

### Western Blot

All Western blot procedures were conducted as previously described [39]. The transfected osteosarcoma cells were collected, and the total protein was extracted using RIPA lysis and extraction buffer (89901, ThermoFisher Scientific). The concentration of extracted protein was measured with a bicinchoninic acid (BCA) protein kit (23225, ThermoFisher Scientific) and 20 μg of the protein lysates was electrophoresed and detached by sodium dodecyl sulfate-polyacrylamide gel electrophoresis (SDS-PAGE, E-IR-R305, Elabscience, China), and quickly transferred into polyvinylidene fluoride (PVDF) membrane (E-IR-R304A, Elabscience). The membrane was blocked using 5% skimmed milk at RT for 2 h and then incubated with the primary antibodies, which included those against TET3 (ab139311, Abcam, UK) and internal control GAPDH (ab8245, Abcam) at 4°C overnight. The membrane was then incubated with the secondary horseradish peroxidase (HRP)-combined antibodies including goat anti-mouse IgG H&L (ab205719, Abcam) and goat anti-rabbit IgG H&L (ab205718, Abcam) at RT for 1 h. All the primary and secondary antibodies were diluted to 1:2000 prior to use. The membrane was subsequently washed with tris-buffer saline tween (TBST, C520009, Sangon Biotech) for three times. The protein band was collected and visualized using an enhanced chemiluminescence (ECL) kit (C510043, Sangon Biotech) and the grey values were further calculated by ImageJ (v. 5.0; Bio-Rad Laboratories, Inc.).

### Statistical Analyses

All experiments were performed three times independently, and data were expressed as mean ± standard deviation (SD). All statistics were analyzed by SPSS 17.0 (IBM Corporation, USA). Statistical significance was determined by one-way ANOVA followed by Bonferroni post hoc test and paired *t* test. Pearson’s correlation analysis was adopted for the correlation analysis between MIR22HG and miR-629-5p. *p*-value below 0.05 was indicative of statistical significance.

## Results

### MiR-629-5p Was the Candidate miRNA which Could Competitively Bind with MIR22HG

**Fig. 1:**
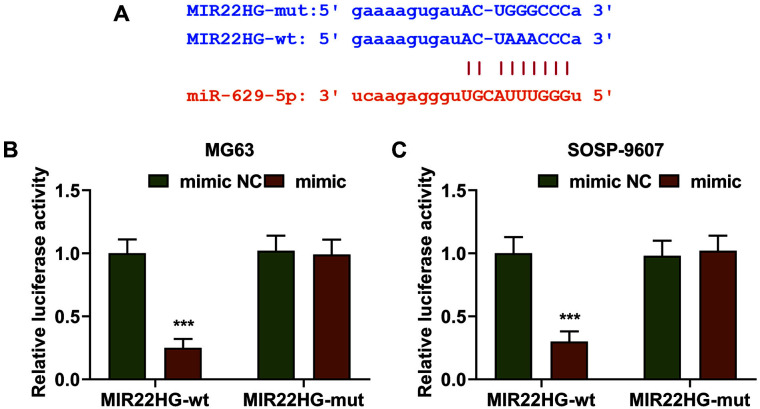
MiR-629-5p could competitively bind with lncRNA MIR22HG. (**A**) The putative binding sites of lncRNA MIR22HG and miR-629-5p were predicted by Starbase (http://www.starbase.sysu.edu.cn). (**B-C**) Dual-luciferase reporter assay confirmed that miR-629-5p could competitively bind with lncRNA MIR22HG. All data were expressed as mean ± standard deviation (SD), which was indicative of three independent tests. ***p < 0.001, vs. mimic NC. miR: microRNA; lncRNA: long non-coding RNA; MIR22HG: MIR22 host gene; NC: negative control; wt: wild-type; mut: mutated.

Previous studies found that MIR22HG plays a tumor suppressor role in a variety of cancers, but its role and mechanism in osteosarcoma are unknown. In this study, we explored the effect of MIR22HG in osteosarcoma. In addition, it has been reported that miR-629-5p promoted osteosarcoma proliferation and migration by targeting caveolin 1 [9]. To confirm the potential interaction between MIR22HG and miR-629-5p, we first downloaded the data concerning the putative binding sites between MIR22HG and miR-629-5p from Starbase (Fig. 1A) and then utilized the dual-luciferase reporter assay to confirm the prediction. As displayed in the results, luciferase activity was decreased in both MG63 and SOSP-9607 cells transfected with the reporter plasmid of MIR22HG-wt and miR-629-5p mimic when compared with those cells transfected with MIR22HG-wt and miR-629-5p mimic NC (Figs. 1B-1C, *p* < 0.001). However, there was no evident change in the luciferase activity of MG63 and SOSP-9607 cells transfected with the reporter plasmid of MIR22HG-mut and miR-629-5p mimic or mimic NC. Taken together, the results suggested that miR-629-5p was the candidate miRNA which could competitively bind with MIR22HG.

### MIR22HG Was Lower Expressed while miR-629-5p Was Higher Expressed in Osteosarcoma Tissues

**Fig. 2:**
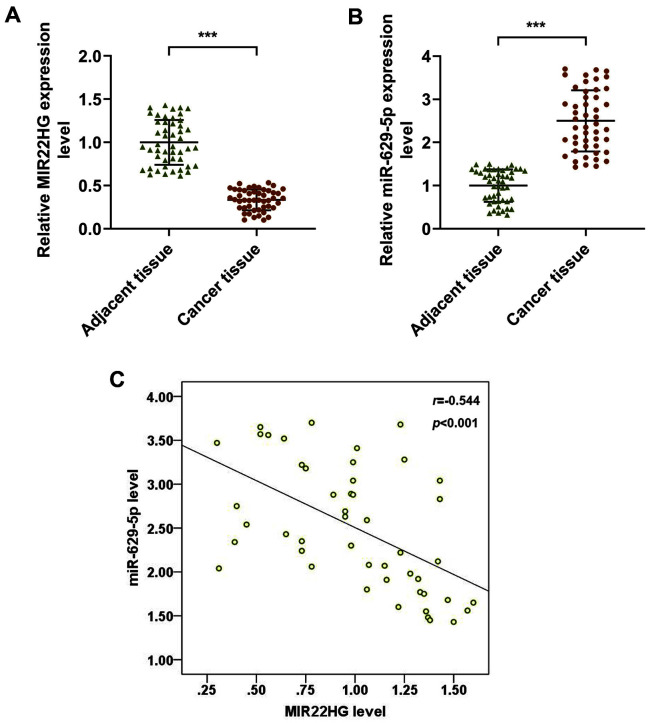
Expressions of MIR22HG and miR-629-5p in osteosarcoma tissue and the correlation of the two genes. (A-B) Relative MIR22HG (**A**) and miR-629-5p (**B**) expressions in osteosarcoma tissue and adjacent tissue were measured by qRT-PCR (*n* = 48 for each tissue). GAPDH (for MIR22HG) and U6 (for miR-629-5p) were used as internal references. (**C**) Pearson’s correlation analysis revealed the negative correlation between MIR22HG and miR-629-5p in osteosarcoma tissues. ***p < 0.001, vs. adjacent tissue. All data were expressed as mean ± standard deviation (SD), which was indicative of three independent tests. qRT-PCR: quantitative real-time polymerase chain reaction.

Subsequently, the expressions of both MIR22HG and miR-629-5p were detected in forty-eight paired osteosarcoma cancer tissues and adjacent tissues. It was found that MIR22HG was lower expressed while miR-629-5p was higher expressed in osteosarcoma tissues as compared with those in adjacent tissues (Figs. 2A-2B, *p* < 0.001). Furthermore, Pearson’s correlation analysis showed that there was a negative correlation between MIR22HG and miR-629-5p expression in osteosarcoma tissues (Fig. 2C, *r* = -0.544, *p* < 0.001).

### Overexpressed MIR22HG Repressed miR-629-5p Expression and the Viability and Proliferation of Osteosarcoma Cells but Enhanced the Apoptosis via Targeting miR-629-5p

**Fig. 3:**
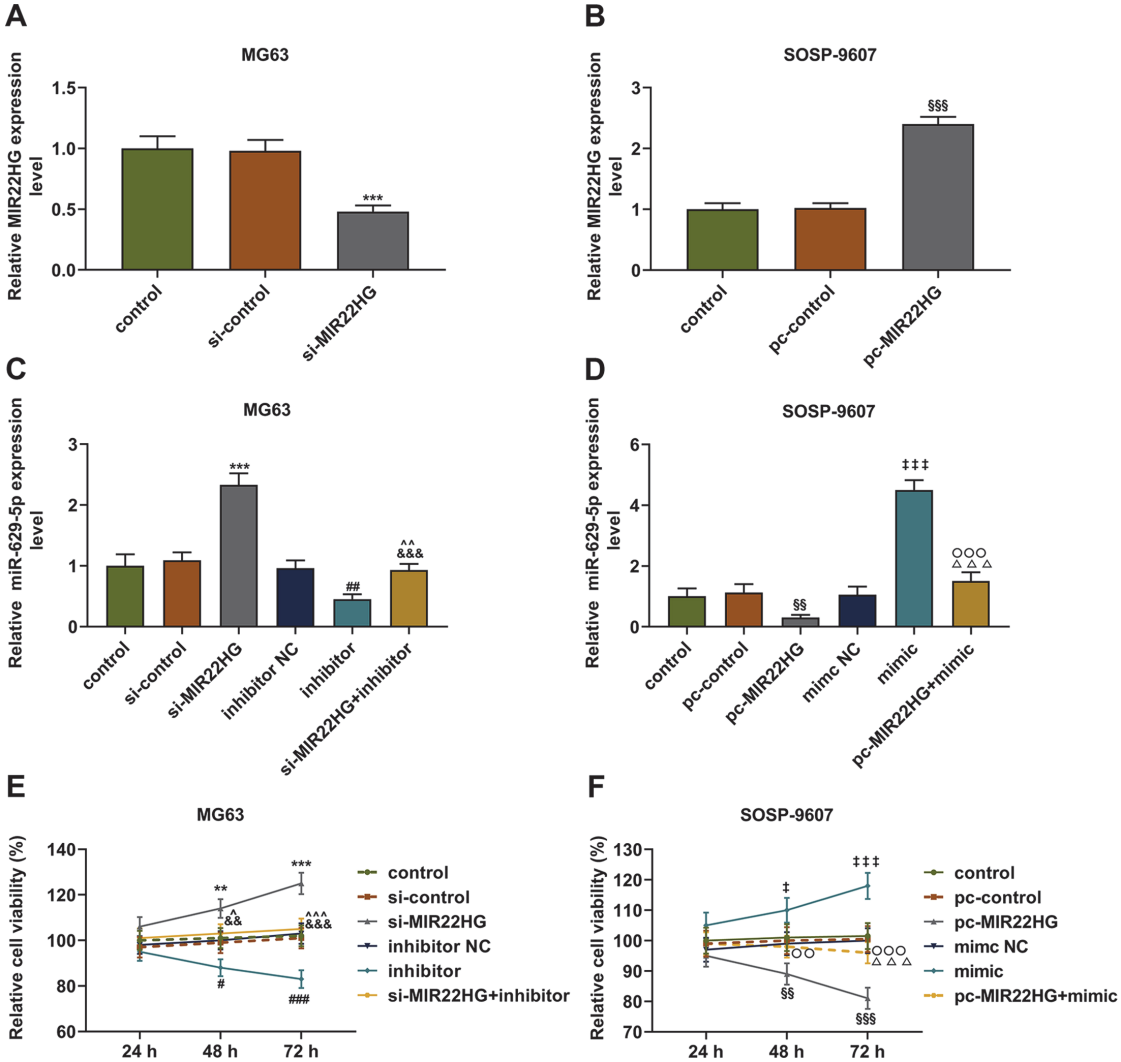
Regulatory effects of MIR22HG and miR-629-5p on miR-629-5p expression and cell viability in osteosarcoma cells. (**A-B**) The transfection efficiency of si-MIR22HG (for MIR22HG silence) and pc-MIR22HG (for MIR22HG overexpression) into osteosarcoma cells MG63 (**A**) and SOSP-9607 (**B**) was determined via qRT-PCR. GAPDH was used as internal control. (**C-D**) Relative miR-629-5p expression following upregulating or downregulating miR-629-5p and silencing or overexpressing MIR22HG in osteosarcoma cells MG63 (**C**) and SOSP-9607 (**D**) was detected by qRT-PCR. U6 was used as internal control. (**E-F**) Relative cell viability of osteosarcoma cells MG63 (**E**) and SOSP-9607 (**F**) following upregulating or downregulating miR-629-5p and silencing or overexpressing MIR22HG at 24, 48, and 72 h was detected by CCK-8 assay. All data were expressed as mean ± standard deviation (SD), which was indicative of three independent tests. **p < 0.01, ***p < 0.001, vs. si-control; ^#^*p* < 0.05, ^##^*p* < 0.01, ^###^*p* < 0.001, vs. inhibitor NC; ^*p* < 0.05, ^^*p* < 0.01, ^^^*p* < 0.001, vs. inhibitor; ^&&^*p* < 0.01, ^&&&^*p* < 0.001, vs. si-MIR22HG; ^§§^*p* < 0.01, ^§§§^*p* < 0.001, vs. pc-control; ^‡^*p* < 0.05, ^‡‡^*p* < 0.01, ^‡‡‡^*p* < 0.001, vs. mimic NC; ^○○^*p* < 0.01, ^○○○^*p* < 0.001, vs. mimic; ^△△^*p* < 0.01, ^△△△^*p* < 0.001, vs. pc-MIR22HG. si-RNA: small interfering RNA; CCK-8: Cell Counting Kit-8.

MG63 and SOSP-9607 cells were transfected with the plasmids (pc-MIR22HG and si-MIR22HG) to increase or decrease MIR22HG, the transfection of which was proved to be successful (Figs. 3A-3B, *p* < 0.001). Then we set out to determine the interaction between MIR22HG and miR-629-5p in osteosarcoma cells and discovered that miR-629-5p inhibitor significantly decreased miR-629-5p expression in MG63 cells, whereas miR-629-5p mimic increased miR-629-5p expression in SOSP-9607 cells (Figs. 3C-3D, *p* < 0.01). Meanwhile, silent MIR22HG raised miR-629-5p expression in MG63 cells, while overexpressed MIR22HG downregulated the miR-629-5p expression in SOSP-9607 cells (Figs. 3C-3D, *p* < 0.01). Besides, miR-629-5p inhibitor abrogated the effect of silent MIR22HG on miR-629-5p expression in MG63 cells, whereas miR-629-5p mimic reversed the effect of overexpressed MIR22HG on miR-629-5p expression in SOSP-9607 cells (Figs. 3C-3D, *p* < 0.01).

Next, the cell viability, proliferation and apoptosis of transfected MG63 and SOSP-9607 cells were detected by CCK-8, colony formation and flow cytometry assays, respectively. The results of CCK-8 assay showed that cell viabilities of MG63 cells at 48 and 72 h were inhibited following the downregulation of miR-629-5p, whereas cell viabilities of SOSP-9607 cells were increased after upregulating miR-629-5p (Figs. 3E-3F, *p* < 0.05). Also, overexpressed MIR22HG was associated with the decreased viability, while silent MIR22HG led to the increase of viability (Figs. 3E-3F, *p* < 0.05). Moreover, it was confirmed that miR-629-5p inhibitor abrogated the effect of silent MIR22HG on the viability of MG63 cells, whereas miR-629-5p mimic reversed the effect of overexpressed MIR22HG on the viability of SOSP-9607 cells (Figs. 3E-3F, *p* < 0.05).

**Fig. 4:**
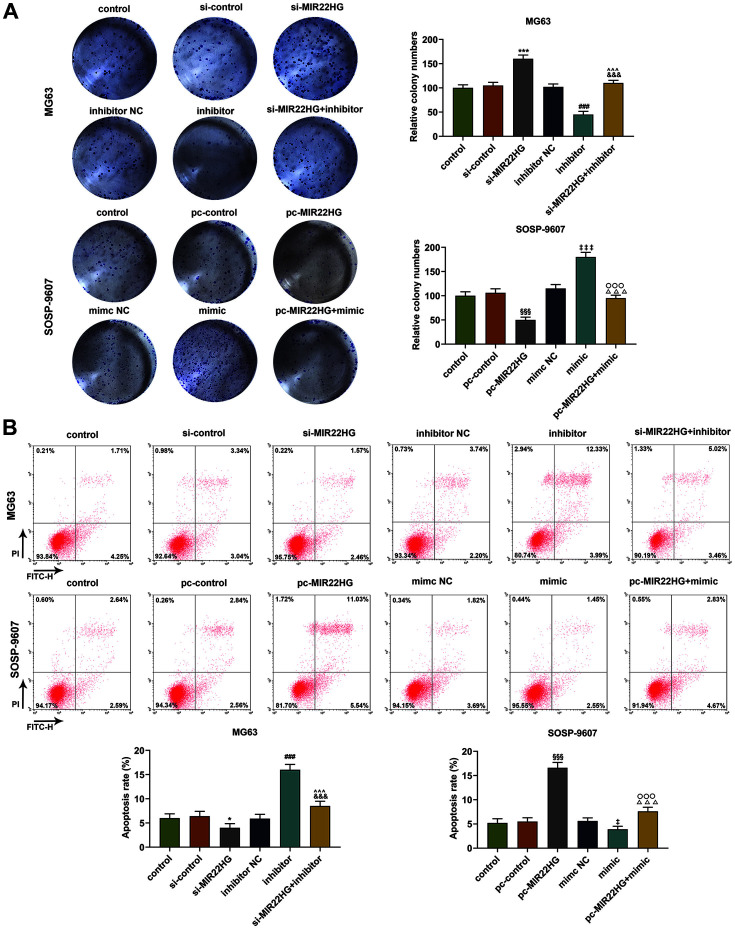
Regulatory effects of MIR22HG and miR-629-5p on the proliferation and apoptosis of osteosarcoma cells. (A-B) Relative colony numbers (**A**) and apoptosis (**B**) in osteosarcoma cells MG63 and SOSP-9607 following upregulating or downregulating miR-629-5p and silencing or overexpressing MIR22HG were quantified with colony formation (**A**) and flow cytometry assays (**B**). All data were expressed as mean ± standard deviation (SD), which was indicative of three independent tests. *p < 0.05, ***p < 0.001, vs. si-control; ^###^*p* < 0.001, vs. inhibitor NC; ^^^*p* < 0.001, vs. inhibitor; ^&&&^*p* < 0.001, vs. si-MIR22HG; ^§§§^*p* < 0.001, vs. pc-control; ^‡^*p* < 0.05, ^‡‡‡^*p* < 0.001, vs. mimic NC; ^○○○^*p* < 0.001, vs. mimic; ^△△△^*p* < 0.001, vs. pc-MIR22HG.

The results of colony formation assay showed that in MG63 cell, the number of colonies formed was increased after the silence of MIR22HG but was decreased by overexpressed MIR22HG (Fig. 4A, *p* < 0.001). Also, the increase of the number of colonies was related to miR-629-5p upregulation, while the decrease was related to miR-629-5p downregulation (Fig. 4A, *p* <0.001). Furthermore, miR-629-5p inhibitor abrogated the effect of silent MIR22HG on the colony formation in MG63 cells, and miR-629-5p mimic reversed the effect of overexpressed MIR22HG on the colony formation in SOSP-9607cells (Fig. 4A, *p* < 0.001).

According to the results from flow cytometry assay, it was found that silent MIR22HG in MG63 cells reduced cell apoptosis, while overexpressed MIR22HG in SOSP-9607 cells showed the opposite result (Fig. 4B, *p* < 0.05). Meanwhile, the apoptosis of SOSP-9607 cells was markedly reduced in miR-629-5p mimic group, whereas the apoptosis of MG63 cells was obviously promoted in miR-629-5p inhibitor group (Fig. 4B, *p* < 0.05). Furthermore, miR-629-5p inhibitor abrogated the effect of silent MIR22HG on the apoptosis of MG63 cells, and miR-629-5p mimic reversed the effect of overexpressed MIR22HG on the apoptosis of SOSP-9607cells (Fig. 4B, *p* < 0.001).

### TET3 Was the Target Gene of miR-629-5p

**Fig. 5:**
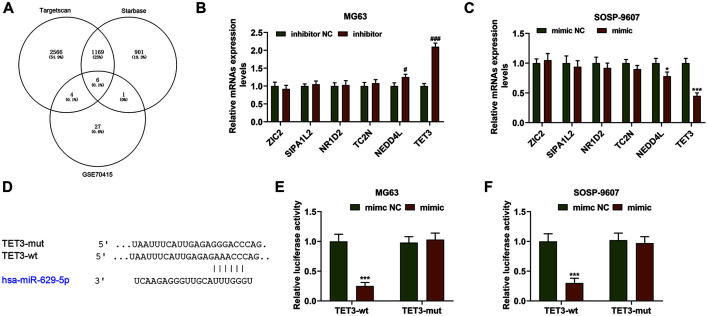
TET3 was the target gene of miR-629-5p. (**A**) A Venn diagram was drawn to sort the candidate gene for miR-629-5p. (**B-C**) Relative mRNA expression of ZIC2, SIPA1L2, NR1D2, TC2N, NEDD4L and TET3 after upregulating or downregulating miR-629-5p in MG63 and SOSP-9607 was detected by qRT-PCR. (**D**) Predicted binding sites of TET3 and miR-629-5p from TargetScan V7.2 (www.targetscan.org/vert_72/). (**E-F**) Dual-luciferase reporter assay confirmed that TET3 was the target gene of miR-629-5p. All data were expressed as mean ± standard deviation (SD), which was indicative of three independent tests. ^#^*p* < 0.05, ^###^*p* < 0.001, vs. inhibitor NC; *p < 0.05, ***p < 0.001, vs. mimic NC. TET3: tet methylcytosine dioxygenase 3.

To further sort the candidate gene for this study, we first downloaded the data from both Starbase and TargetScan V7.2, and retrieved the dataset GSE70415 (the mRNA and miRNA expression from human osteosarcoma) from GEO. All the results were then summarized and displayed in a Venn diagram, as described in Fig. 5A. Subsequently, six common genes (ZIC2, SIPA1L2, NR1D2, TC2N, NEDD4L, and TET3) were sorted. In addition, the effect of miR-629-5p on these candidate genes was detected, and the result showed that miR-629-5p significantly inhibited the expression of TET3, but had no significant effect on the expression of other candidate genes except for NEDD4L (Figs. 5B-5C). Moreover, TET3, as a target gene of miR-135b, participated in the recurrence and lung metastasis of osteosarcoma [15]. In light of this discovery, we speculated that TET3 was also the target gene of miR-629-5p, which was confirmed by TargetScan (Fig. 5D). Besides, the dual-luciferase reporter assay also further verified this targeting relationship, and illustrated that the luciferase activity of both MG63 and SOSP-9607 cells transfected with the reporter plasmid of TET3-wt and miR-629-5p mimic was decreased in comparison with the luciferase activity of those cells transfected with TET3-wt and miR-629-5p mimic NC (Figs. 5E-5F, *p* < 0.001). However, there was no evident change in the luciferase activity of MG63 and SOSP-9607 cells transfected with the reporter plasmid of TET3-mut and miR-629-5p mimic or mimic NC. It could therefore be summarized that TET3 was the target gene of miR-629-5p.

### TET3 Reversed the Effects of miR-629-5p on TET3 Expression, Cell Viability, Proliferation and Apoptosis of Osteosarcoma Cells

**Fig. 6:**
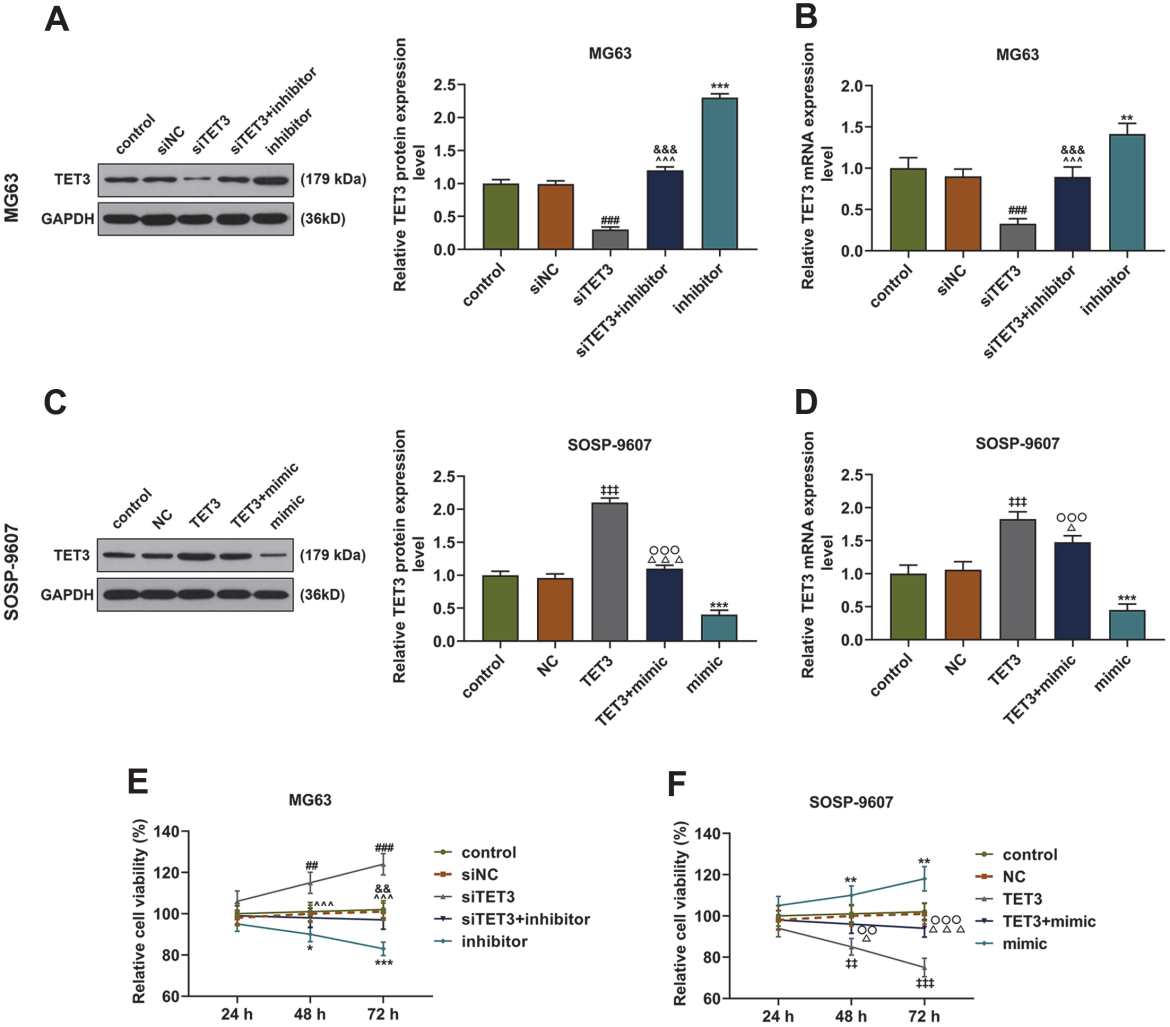
Regulatory effects of TET3 and miR-629-5p on TET3 expression and the viability of osteosarcoma cells. (A-D) Relative protein and mRNA expression of TET3 after upregulating or downregulating miR-629-5p and silencing or overexpressing TET3 in MG63 (**A-B**) and SOSP-9607 (**C-D**) cells were quantified by Western blot and qRT-PCR, and GAPDH was used as internal control. (**E-F**) Relative cell viability of MG63 (**E**) and SOSP-9607 (**F**) cells after upregulating or downregulating miR-629-5p and silencing or overexpressing TET3 at 24, 48 and 72 hours was quantified by CCK-8 assay. All data were expressed as mean ± standard deviation (SD), which was indicative of three independent tests. ^##^*p* < 0.01, ^###^*p* < 0.001, vs. siNC; *p < 0.05, **p < 0.01, ***p < 0.001, vs. control; ^&^*p* < 0.05, ^&&^*p* < 0.01, ^&&&^*p* < 0.001, vs. inhibitor; ^^^*p* < 0.001, vs. siTET3; ^‡‡^*p* < 0.01, ^‡‡‡^*p* < 0.001, vs. NC; ^△^*p* < 0.05, ^△△△^*p* < 0.001, vs. TET3; ^○○^*p* < 0.01, ^○○○^*p* < 0.001, vs. mimic.

Then we set out to determine the interaction between miR-629-5p and TET3 on transfected osteosarcoma cells, and the effects of miR-629-5p and TET3 on the expression of TET3. The results indicated that the TET3 expression was increased by downregulation of miR-629-5p in MG63 cells, and silencing TET3 induced the downregulation of TET3 (Figs. 6A-6B, *p* < 0.01). However, in SOSP-9607 cells, TET3 overexpression was associated with the elevation of TET3 expression, whereas miR-629-5p repressed TET3 expression (Figs. 6C-6D, *p* < 0.001). Therefore, it can be further concluded that TET3 could abolish the effects of miR-629-5p on TET3 expression in both MG63 and SOSP-9607 cells (Figs. 6A-6D, *p* < 0.05). In addition, we also detected the viability of transfected osteosarcoma cells MG63 and SOSP-9607. According to the results, silent TET3 raised the viability of osteosarcoma cells, whereas the overexpressed TET3 acted conversely (Figs. 6E-6F, *p* < 0.01). The results also revealed that downregulating miR-629-5p expression in MG63 cells repressed the viability, whereas upregulating miR-629-5p expression in SOSP-9607 cells led to increased viability (Figs. 6E-6F, *p* < 0.05). Obviously, TET3 abolished the effects of miR-629-5p on the viability of transfected osteosarcoma cells (Figs. 6E-6F, *p* < 0.05).

**Fig. 7:**
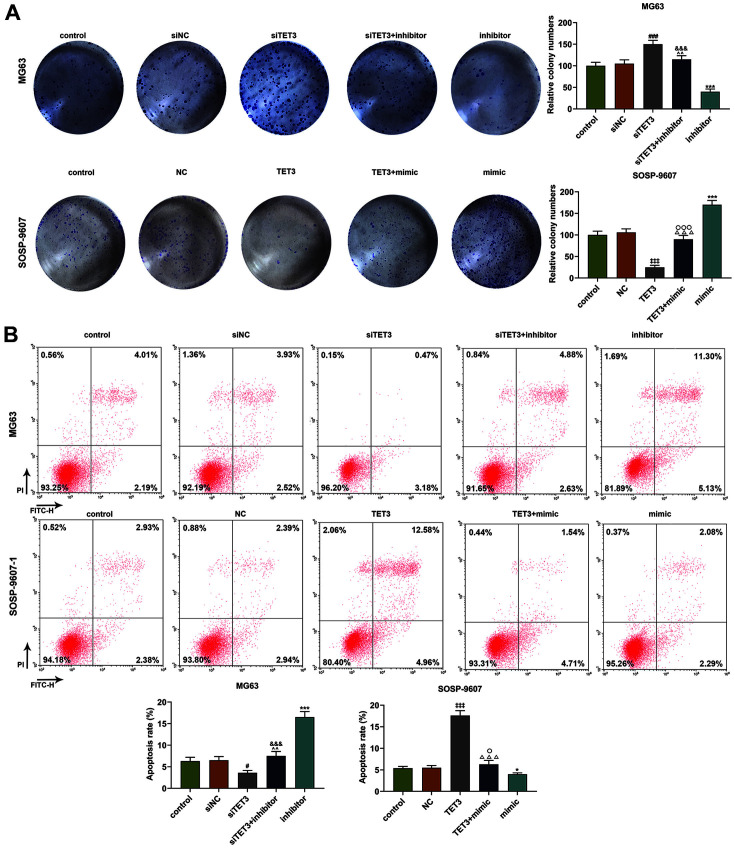
Regulatory effects of TET3 and miR-629-5p on the proliferation and apoptosis of osteosarcoma cells. (**A**) Relative colony numbers formed in osteosarcoma cells MG63 and SOSP-9607 after upregulating or downregulating miR- 629-5p and silencing or overexpressing TET3 were determined with colony formation assay. (**B**) Relative cell apoptosis rates in osteosarcoma cells MG63 and SOSP-9607 after upregulating or downregulating miR-629-5p and silencing or overexpressing TET3 were detected with flow cytometry assay. All data were expressed as mean ± standard deviation (SD), which was indicative of three independent tests. ^#^*p* < 0.05, ^###^*p* < 0.001, vs. siNC; *p < 0.05, **p < 0.01, ***p < 0.001, vs. control; ^^*p* < 0.01, vs. siTET3; ^&&&^*p* < 0.001, vs. inhibitor; ^‡‡‡^*p* < 0.001, vs. NC; ^△△△^*p* < 0.001, vs. TET3; ^○^*p* < 0.05, ^○○○^*p* < 0.001, vs. mimic.

According to the results from colony formation, the number of colonies formed was increased after the silence of TET3 in MG63 cells. Besides, the downregulation of miR-629-5p was associated with the decreased colony formation, which was reversed by downregulating TET3 (Fig. 7A, *p* < 0.01). However, in SOSP-9607 cells, overexpressed TET3 reduced the number of colonies formed, whereas upregulation of miR-629-5p did the opposite. The promotive effect of miR-629-5p mimic on the cell proliferation was reversed by overexpressed TET3 (Fig. 7A, *p* < 0.001). Based on the results of flow cytometry, the apoptosis was found to be declined following the silence of TET3; however, miR-629-5p downregulation enhanced the apoptosis, which was further found to be reversed by the silence of TET3 (Fig. 7B, *p* < 0.05). In addition, overexpressed TET3 in SOSP-9607 cells promoted the cell apoptosis, while upregulation of miR-629-5p acted oppositely. Moreover, the effect of upregulation of miR-629-5p on apoptosis was confirmed to be reversed by the overexpressed TET3 (Fig. 7B, *p* < 0.05).

## Discussion

Osteosarcoma, defined as a cancerous tumor residing in bone, is the most common histological form of primary bone cancer. Due to the lack of effective diagnosis and clinical treatment for patients with this disease, it is therefore of great urgency and significance to develop an effective clinical treatment [21, 16]. Increasing evidence has suggested that ncRNAs, including lncRNAs and miRNAs, play significant roles in the progression of osteosarcoma and therefore could be used as potential biomarkers for osteosarcoma [5]. During the tumorigenesis and development, the participation of the tumor microenvironment (TME) is not negligible, and increasing discoveries have suggested that the MSCs, as the components of TME, play a pivotal role in the mediation and proliferation in multiple tumors, including osteosarcoma [42]. The TME has been found to elicit different effects on the virgin MSCs, which can be recruited by some cytokines to the tumor sites and further stimulated by the paracrine network and finally undergo a series of functional transformations [8]. Meanwhile, the pathogenesis of osteosarcoma has been investigated and shown to be generally related to the alternation of genes associated with regulation of the cell cycle and apoptosis [25]. Prior publications have additionally highlighted the impact of MIR22HG on the proliferation and apoptosis of a variety of human malignancies, along with reports suggesting that MIR22HG can either act as a tumor suppressor or promoter in lung cancer, glioblastoma and hepatocellular carcinoma (HCC), for instance [10, 11, 31, 41]. However, the molecular mechanism of MIR22HG in osteosarcoma needs to be further elucidated in detail, as there has been little discussion. As far as we are concerned, the role of MIR22HG in osteosarcoma is confirmed in this study for the first time. Specifically, overexpressed MIR22HG is an lncRNA low-expressed in osteosarcoma, which is associated with the suppressed viability and proliferation and the promoted apoptosis of osteosarcoma cells, the effects of which are associated with the regulation on miR-629-5p/TET3 axis. Consequently, it can be assumed that overexpressed MIR22HG might suppress the viability and proliferation but promote apoptosis in osteosarcoma cells.

It has also been uncovered that lncRNAs share the same complementary binding sites with target miRNA as the competing endogenous RNAs (ceRNAs), which thereby affects and regulates the expressions of both mRNAs and target genes, in addition to the discovery providing for the participation and regulatory role of both lncRNA-miRNA-mRNA axis and miRNAs in osteosarcoma [35, 40, 43]. MiR-629 has been shown to be elevated in osteosarcoma, which is associated with the promotion of the cell proliferation, migration and invasion of osteosarcoma, in addition to the discoveries suggesting its participation in other cancers, such as breast cancer, acute lymphoblastic leukemia and clear cell renal carcinoma [2, 18, 33, 34]. In addition, increasing evidence has suggested that MIR22HG can bind to other miRNAs so as to elicit its functions on different cancers [32]. Nevertheless, to the best of our knowledge, there’s no discussion on the interaction between MIR22HG and miR-629-5p in osteosarcoma. In the current study, MIR22HG shares the binding sites with miR-629-5p, a miRNA whose expression is upregulated in osteosarcoma, and further results from Pearson’s correlation test suggests the negative correlation between miR-629-5p and MIR22HG. Furthermore, it can be concluded that the effects of MIR22HG on osteosarcoma cells are achieved by targeting miR-629-5p, which further completes the discoveries on the roles of both MIR22HG and miR-629-5p in osteosarcoma. Nevertheless, the detailed molecular mechanism waits to be additionally addressed.

MiRNAs are a series of small RNA molecules which have been discovered to base-pair to the 3’-untranslated region (UTR) of target mRNAs thus downregulating related gene expression [30]. TET proteins are seen to play a major role in the maintenance of the fidelity of DNA methylation patterns via the mediation on demethylation, and have also been elucidated to be related to some tumors, including osteosarcoma [15, 22, 26]. In addition, TET3 has been suggested as the target gene of miRNAs, miR-135b to be exact [15]. This discovery made us wonder whether the effects of miR-629-5p on osteosarcoma could be achieved by targeting TET3. From the present study, it can be confirmed that TET3 is the target gene of miR-629-5p, and TET3 overexpression represses the viability and proliferation and enhances the apoptosis of osteosarcoma cells. Meanwhile, further experiments suggested that TET3 abolishes the effects of miR-629-5p on the viability, proliferation and apoptosis of osteosarcoma cell, which not only provides the evidence that the effects of miR-629-5p on the viability, proliferation and apoptosis are achieved by targeting TET3, but also further completes the role of TET3 and its interaction with miRNAs in osteosarcoma.

However, there are still some limitations to be addressed in this study as all of our results are concluded based on the experiments in vitro, and therefore an equivalent validation in vivo is thus required to complete the results.

In conclusion, the discoveries in this study unveil the participation of lncRNA MIR22HG in osteosarcoma, and demonstrate that overexpressed MIR22HG represses the viability and proliferation but promotes the apoptosis of osteosarcoma cells via the miR-629-5p/TET3 axis. This study proves the implication of MIR22HG in osteosarcoma, suggesting that MIR22HG may be used as a potential suppressor for osteosarcoma in clinical practice in the future.

## Figures and Tables

**Table 1:** Sequences for transfection.

Gene	Sequence (5’-3’)
si-MIR22HG sense obligo	UUGUUUGAGCCUUCUACUCCU
si-MIR22HG antisense obligo	GAGUAGAAGGCUCAAACAACC
si-control sense obligo	UUGUUAGCCUUUCUACUGCCU
si-control antisense obligo	GAGGCUCGAAGCAAACAACUA
miR-629-5p mimic	UGGGUUUACGUUGGGAGAACU
miR-629-5p inhibitor	AGUUCUCCCAACGUAAACCCA
miR-629-5p mimic NC	UGGGUUUUGACGUUGGAGAAC
miR-629-5p inhibitor NC	AGUCCCAACGUCCCUCAAAAU
siTET3 sense obligo	UCACAUUUUCGCAGUUUGCAG
siTET3 antisense obligo	GCAAACUGCGAAAAUGUGAGG
siNC sense obligo	UUGUUUUCGCAGCAGUCACAU
siNC antisense obligo	GCAAAUGGCGUAGCUAGAAGA

**Table 2:** Primers for qRT-PCR.

Gene	Primers (5’-3’)	

	Forward	Reverse
MIR22HG	CCTGTATCTGAGACCCTTAG	AGTAGCAGCAGTTAGAATCC
miR-629-5p	ACGTTGGGAGAACTGTCGTA	GTATCCAGTGCGTGTCGTGG
TET3	CCTTTATGACTTCCCTCAG	CTTGAGAAGCCCTACTTTT
U6	CTCGCTTCGGCAGCACATATACT	ACGCTTCACGAATTTGCGTGTC
GAPDH	TTTTTGGTTTTAGGGTTAGTTAGTA	AAAACCTCCTATAATATCCCTCCTC
